# Long-Term Bacterial and Fungal Dynamics following Oral Lyophilized Fecal Microbiota Transplantation in Clostridioides difficile Infection

**DOI:** 10.1128/mSystems.00905-20

**Published:** 2021-02-02

**Authors:** Craig Haifer, Sudarshan Paramsothy, Thomas J. Borody, Annabel Clancy, Rupert W. Leong, Nadeem O. Kaakoush

**Affiliations:** a Concord Clinical School, The University of Sydney, Sydney, Australia; b Gastroenterology Department, Concord Repatriation General Hospital, Sydney, Australia; c Faculty of Medicine and Health Sciences, Macquarie University, Sydney, Australia; d Centre for Digestive Diseases, Sydney, Australia; e School of Medical Sciences, University of New South Wales, Sydney, Australia; Mayo Clinic

**Keywords:** fecal microbiota transplantation, *Clostridioides difficile* infection, mycobiome, microbiome

## Abstract

Oral lyophilized fecal microbiota transplantation (FMT) is effective in recurrent Clostridioides difficile infection (CDI); however, limited data exist on its efficacy in primary CDI and long-term microbial engraftment. Patients with primary or recurrent CDI were prospectively enrolled to receive oral FMT. Changes in the bacterial and fungal communities were characterized prior to and up to 6 months following treatment. A total of 37 patients with CDI (15 primary, 22 recurrent) were treated with 6 capsules each containing 0.35-g lyophilized stool extract. A total of 33 patients (89%) had sustained CDI cure, of whom 3 required a second course. There were no safety signals identified. FMT significantly increased bacterial diversity and shifted composition toward donor profiles in responders but not in nonresponders, with robust donor contribution observed to 6 months following FMT (*P* < 0.001). Responders showed consistent decreases in *Enterobacteriaceae* and increases in *Faecalibacterium* sp. to levels seen in donors. Mycobiome profiling revealed an association with FMT failure and increases in one *Penicillium* taxon, as well as coexclusion relationships between *Candida* sp. and bacterial taxa enriched in both donors and responders. Primary CDI was associated with more robust changes in the bacterial community than those with recurrent disease. Oral FMT leads to durable microbial engraftment in patients with primary and recurrent CDI, with several microbial taxa being associated with therapy outcome. Novel coexclusion relationships between bacterial and fungal species support the clinical relevance of transkingdom dynamics.

**IMPORTANCE** Clostridioides difficile infection (CDI) is a substantial health concern worldwide, complicated by patterns of increasing antibiotic resistance that may impact primary treatment. Orally administered fecal microbiota transplantation (FMT) is efficacious in the management of recurrent CDI, with specific bacterial species known to influence clinical outcomes. To date, little is known about the efficacy of FMT in primary CDI and the impact of the mycobiome on therapeutic outcomes. We performed matched bacterial and fungal sequencing on longitudinal samples from a cohort of patients treated with oral FMT for primary and recurrent CDI. We validated many bacterial signatures following oral therapy, confirmed engraftment of donor microbiome out to 6 months following therapy, and demonstrated coexclusion relationships between Candida albicans and two bacterial species in the gut microbiota, which has potential significance beyond CDI, including in the control of gut colonization by this fungal species.

## Abstract (precis)

Clostridioides difficile infection (CDI) is a substantial health concern worldwide, complicated by patterns of increasing antibiotic resistance that may impact primary treatment. Orally administered fecal microbiota transplantation (FMT) is efficacious in the management of recurrent CDI, with specific bacterial species known to influence clinical outcomes.

## INTRODUCTION

Recurrence following Clostridioides difficile infection (CDI) is common following antibiotic therapy, usually within 30 days of treatment cessation (1). Fecal microbiota transplantation (FMT) is now recognized as the preferred treatment in recurrent CDI. It alleviates the microbial imbalance seen in the disease and reduces the risk of recurrences compared with antibiotic therapy (2, 3). Lyophilization uses a freeze-drying technique that allows encapsulation of FMT and dose standardization to 10^10^ or more bacteria per capsule, along with maintenance of bacterial viability for greater than 7 months following production (4, 5). Oral lyophilized FMT is effective in the treatment of recurrent CDI with engraftment of donor microbial species in the recipient (4). While existing evidence suggests particular bacterial species, such as those involved in carbohydrate fermentation to short-chain fatty acids, are important in mediating FMT outcomes (6–9), information on the role of the mycobiome in disease development and FMT therapeutic outcomes remains limited. Furthermore, little is known about persistent microbial engraftment beyond 12 weeks and whether reduction in donor microbial contribution in the recipient over time will lead to recurrence of disease.

The growing resistance of C. difficile to antibiotics is also a significant health concern (10) since currently recommended therapeutic guidelines for primary CDI involve antibiotic therapy. The role of FMT in the treatment of primary CDI is not established; however, early evidence suggests comparable efficacy with antibiotics (11–13), indicating that it could serve as an alternate therapeutic strategy.

Here, we report the outcomes of a prospectively enrolled “real world” cohort of consecutive patients with primary and recurrent CDI treated with oral lyophilized FMT and investigate how resultant changes in the bacterial and fungal communities can impact therapeutic outcomes.

## RESULTS

### Orally administered lyophilized FMT is safe and durably effective for treating CDI.

A total of 37 patients received oral FMT for CDI (15 primary CDI, 22 recurrent CDI) with a median follow up of 17 weeks (range, 4 to 26). The mean ± standard deviation (SD) age was 42.3 ± 19.8 years. Twenty (54%) patients were male. Patient and disease characteristics are outlined in Table 1. In total, 33 (89.2%) patients had sustained clinical and biochemical cure to the end of follow up, of whom 3 (9.1%) required a second course of oral FMT. Sustained CDI cure rates were similar between primary and recurrent disease (13/15 [86.7%] versus 20/22 [90.9%]), although the study was not powered to detect a difference between the indications.

**TABLE 1:** Baseline patient characteristics

Parameter	Primary CDI (*n* = 15)	Recurrent CDI (*n* = 22)
Patient characteristics		
Age, mean (SD)	36.7 (16.5)	46.2 (21.4)
Male sex, *n* (%)	10 (66.7)	10 (45.5)
Disease characteristics		
No. of recurrences, mean (SD)	0	1.8 (1.1)
Previous antibiotic therapy for CDI, *n* (%)		
Metronidazole	0	10 (45.4)
Vancomycin	0	19 (86.3)

Minor gastrointestinal adverse events occurred in 45% of patients, including nausea, abdominal discomfort, constipation, and diarrhea; all events were self-limiting. One patient had fevers following treatment that resolved without intervention. There were no serious adverse events attributable to oral FMT therapy. No patients required colectomy during the follow-up period.

### Bacterial community in responders to FMT appear to be more susceptible to manipulation.

**FIG 1:**
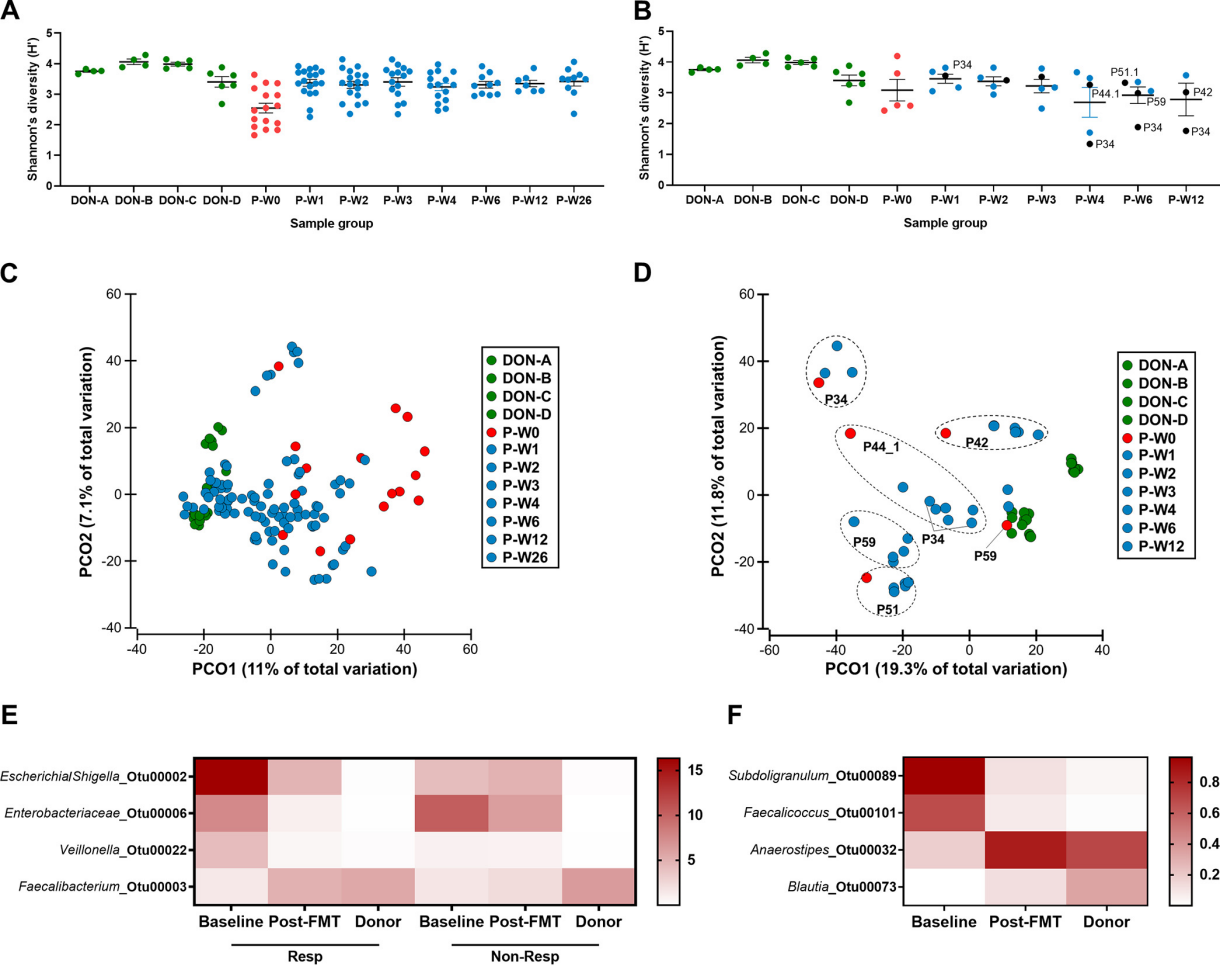
Changes to the bacterial communities. Both primary and recurrent CDI are included. Two patients did not provide baseline samples. (A) Shannon’s diversity (H´) indices in donors and responders to FMT. Significance was tested using ANOVA with Tukey’s multiple-comparison test, and P-W0 was found to be statistically significantly different from all other groups. No other comparisons were significant. (B) Shannon’s diversity (H´) index in donors and nonresponders to FMT. Patient samples at recurrence were labeled in black and with patient number. P34 had persistent disease. Significance was tested using ANOVA with Tukey’s multiple-comparison test, and P-W4 was found to be significantly different from donors B and C. No other comparisons were significant. (C) Principal-coordinate analysis of responders to FMT and donors. Bray-Curtis resemblance matrix was generated from square-root-transformed relative abundances of bacterial OTUs. All patient subgroups (P-) were significantly different from the donors (DON) when tested using pairwise PERMANOVA (*P* < 0.005 for all). P-W0 was significantly different from all other patient subgroups (*P* < 0.006 for all). No other comparisons were significant. ANOSIM confirmed the pairwise PERMANOVA results. (D) Principal-coordinate analysis of nonresponders to FMT and donors. Bray-Curtis resemblance matrix was generated from square-root-transformed relative abundances of bacterial OTUs. Dotted lines indicate samples corresponding to the same patient unless otherwise indicated. All patient subgroups (P-) were significantly different from the donors (DON) when tested using pairwise PERMANOVA (*P* < 0.004 for all). No other comparisons were significant. ANOSIM confirmed the pairwise PERMANOVA results. (E) Heatmap of mean relative abundance of bacterial OTUs found to be consistently significantly different between responders’ baseline and all post-FMT samples as well as responders’ baseline and donor samples. OTUs were not found to be significantly different in nonresponders but were included for comparison. Tests were performed using LEfSe, and a strict cutoff LDA score of >4 and *P* value of <0.05 were applied. (F) Heatmap of mean relative abundance of bacterial OTUs found to be consistently significantly different between nonresponders’ baseline and all post-FMT samples as well as nonresponders’ baseline and donor samples. Tests performed using LEfSe and a cutoff LDA score of >3.5 and *P* value of <0.05 were applied.

Twenty-three patients (62.2% of patient cohort) and 4 donors (100% of donors) provided a total of 166 fecal samples. Patients (18 responders and 5 nonresponders) were sampled from baseline up to week 26 following FMT therapy when possible. Baseline samples from two patients could not be obtained. Analysis of the bacterial community of responders and nonresponders to FMT as well as their donors identified key differences in the effect of FMT on these groups. In patients who responded to FMT, there was a significantly lower baseline alpha diversity than in donors and a significant increase in alpha diversity measures to levels observed in donors following FMT (Fig. 1A; see Fig. S2A and B in the supplemental material). These findings were not replicated in the nonresponders (Fig. 1B; Fig. S2C and D), with a sustained difference in species richness between donors and recipients throughout the examined time points (Fig. S2C). There was a sustained but incomplete shift in bacterial composition in responders toward the donors, with all post-FMT samples being significantly different from baseline samples (*P* < 0.005 for all, permutational multivariate analysis of variance [PERMANOVA]) as well as donor samples (*P* < 0.006 for all, PERMANOVA) (Fig. 1C). This again was not replicated in nonresponders, with one patient even showing a shift away from donors (Fig. 1D). The most prominent discriminatory factor between responders and nonresponders was the significant changes in relative abundances of dominant operational taxonomic units (OTUs) toward donor levels in responders (Fig. 1E), which was not observed in nonresponders (Fig. 1E and F). This result corresponded to the depletion of *Enterobacteriaceae* and enrichment of *Faecalibacterium* sp. (linear discriminant analysis [LDA] score of >4 and *P* < 0.05) (Fig. 1E). A possible explanation for the resistance to beneficial microbiome manipulation in nonresponders was their higher levels of *Ruminococcaceae* at baseline and robust enrichment of *Bifidobacterium* sp. (see Fig. S3 in the supplemental material).

10.1128/mSystems.00905-20.1 FIG S1 Minimal amplification and identification of fungal taxa in negative controls. Download FIG S1, TIF file, 0.3 MB. Copyright © 2021 Haifer et al. 2021 Haifer et al. This content is distributed under the terms of the Creative Commons Attribution 4.0 International license.

10.1128/mSystems.00905-20.2 FIG S2 Alpha diversity measures. (A) Species richness (d) in donors and responders to FMT. Significance was tested using ANOVA with Tukey’s multiple-comparison test, and P-W0 was found to be statistically significantly different from all other groups. Donors B and C were also significantly different from all other patient subgroups. (B) Species evenness (J´) in donors and responders to FMT. Significance was tested using ANOVA with Tukey’s multiple-comparison test, and P-W0 was significantly different from donors A, B, and C as well as P-W1, 2, 3, 4, and 26. (C) Species richness (d) in donors and non-responders to FMT. Significance was tested using ANOVA with Tukey’s multiple-comparison test, and donors B and C were significantly different from all patient subgroups. No other comparisons were significant. (D) Species evenness (J´) in donors and non-responders to FMT. Significance was tested using ANOVA with Tukey’s multiple-comparison test, and no comparisons were found to be significant. Download FIG S2, TIF file, 1.1 MB. Copyright © 2021 Haifer et al. 2021 Haifer et al. This content is distributed under the terms of the Creative Commons Attribution 4.0 International license.

10.1128/mSystems.00905-20.3 FIG S3 Comparison of bacterial component of the microbiome between responders and non-responders. (A) Alpha diversity measures at baseline (P-W0) and week 1 of FMT (P-W1) for responders (-Resp) and non-responders (-Non). Significance was tested using ANOVA with Tukey’s multiple-comparison test. (B) Heatmap of mean relative abundance of bacterial OTUs found to be significantly different between responders and non-responders at baseline and week 1 FMT. Tests were performed using LEfSe and a cutoff of LDA score of >4 and *P* value of <0.05 were applied. Download FIG S3, TIF file, 0.5 MB. Copyright © 2021 Haifer et al. 2021 Haifer et al. This content is distributed under the terms of the Creative Commons Attribution 4.0 International license.

### Fungal richness and *Penicillium* sp. were associated with FMT failure.

**FIG 2:**
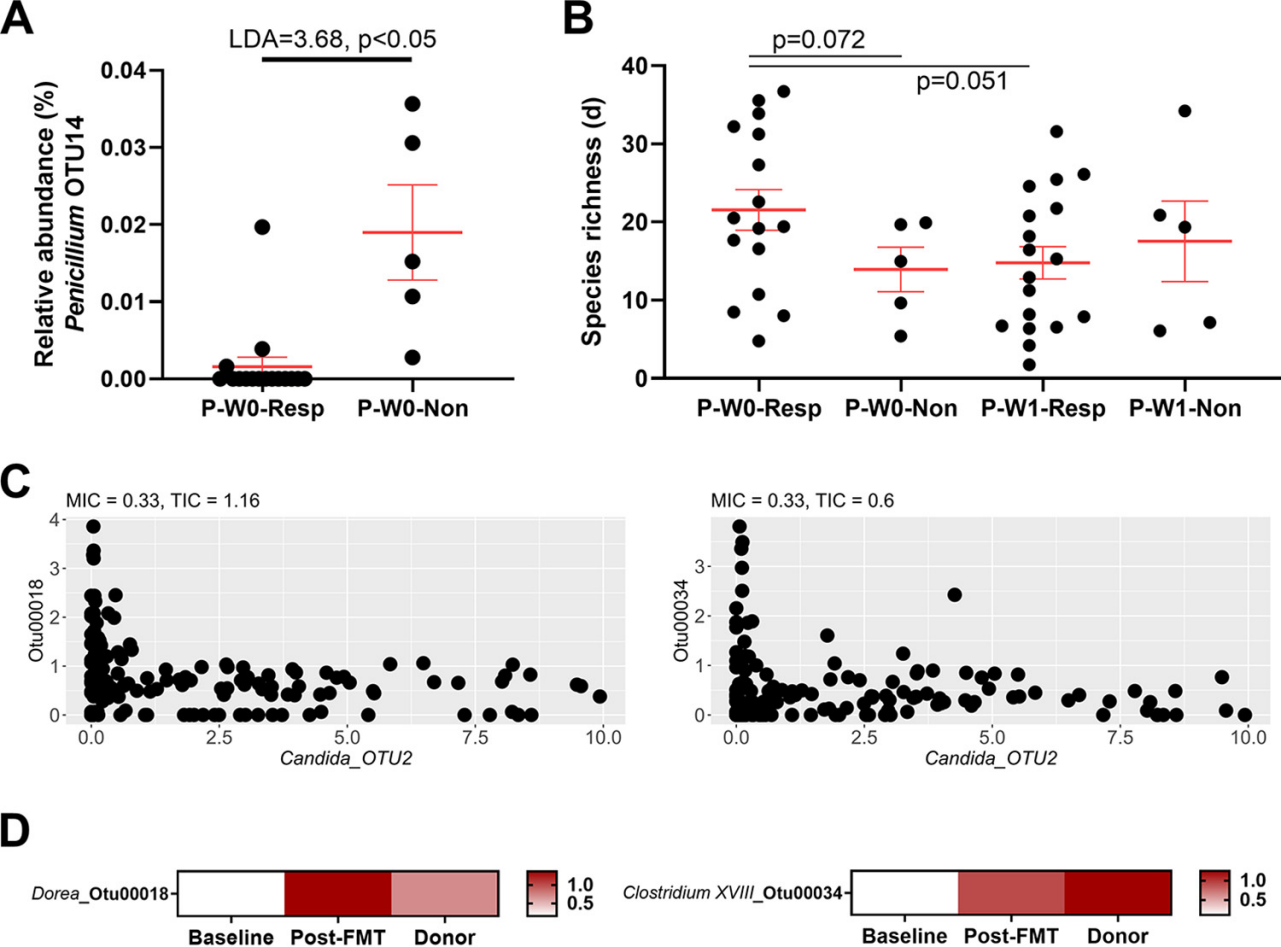
Mycobiome diversity and composition. (A) Relative abundance of *Penicillium* OTU14 which was significantly different between responders and nonresponders at baseline. Testing was performed using LEfSe (LDA score, 3.68; *P* < 0.05). (B) Species richness (d) at baseline (P-W0) and week 1 of FMT (P-W1) for responders (-Resp) and nonresponders (-Non). Significance was tested using Welch’s *t* test for each of the two comparisons reported. (C) Coexclusion relationships between *Candida* OTU2 with similarity to Candida albicans and two bacterial taxa (OTU18 and OTU34). Nonparametric relationships were identified using the MINE framework. OTU18 was classified to *Dorea*, and OTU34 was classified to *Clostridium* XVIII. (D) Heatmaps of mean relative abundances of bacterial OTU18 and OTU34 across responders’ baseline and post-FMT samples as well as donor samples.

Differences in the mycobiome between responders and nonresponders were examined. While no difference was observed in β-diversity (composition) metrics (see Fig. S4 in the supplemental material), nonresponders at baseline had a significantly higher relative abundance of one *Penicillium* taxon than responders (LDA score of 3.68 and *P* < 0.05) (Fig. 2A). No other taxon initially identified as different using linear discriminant analysis effect size (LEfSe) survived sensitivity analysis (see Fig. S5 in the supplemental material). There was a borderline nonsignificant (*P* = 0.072) difference in baseline fungal species richness in responders compared with that of nonresponders (Fig. 2B), with higher richness in responders that decreased with FMT (*P* = 0.051). Other alpha diversity metrics were not significantly different between responders and nonresponders (see Fig. S6 in the supplemental material).

10.1128/mSystems.00905-20.4 FIG S4 Beta diversity within the mycobiome for responders and non-responders. Principal-coordinate analysis of Bray-Curtis resemblance matrix generated from square-root-transformed relative abundances of fungal OTUs. No groups were found to be significantly different from each other using pairwise PERMANOVA. Download FIG S4, TIF file, 0.7 MB. Copyright © 2021 Haifer et al. 2021 Haifer et al. This content is distributed under the terms of the Creative Commons Attribution 4.0 International license.

10.1128/mSystems.00905-20.5 FIG S5 Relative abundance of fungal taxa found to significantly different between responders and non-responders either at baseline or week 1 FMT. Tests were performed using LEfSe. Unlike *Penicillium* OTU14, these comparisons were found to be driven by one outlier and did not survive sensitivity analysis. Download FIG S5, TIF file, 0.5 MB. Copyright © 2021 Haifer et al. 2021 Haifer et al. This content is distributed under the terms of the Creative Commons Attribution 4.0 International license.

10.1128/mSystems.00905-20.6 FIG S6 Alpha diversity measures within the mycobiome. (A) Responders. (B) Non-responders. Significance was tested using ANOVA with Tukey’s multiple-comparison test, and no comparison was significant. Download FIG S6, TIF file, 1.1 MB. Copyright © 2021 Haifer et al. 2021 Haifer et al. This content is distributed under the terms of the Creative Commons Attribution 4.0 International license.

The relationship between the fungal and bacterial communities in this cohort was then assessed. A significant correlation was identified between the resemblance matrices of the two biomes (Spearman’s rho, 0.139; *P* = 0.005), and this persisted even if the cohort was stratified into patients (Spearman’s rho, 0.128; *P* = 0.011) and donors (Spearman’s rho, 0.222; *P* = 0.007), as well as if patients were stratified by baseline (Spearman’s rho, 0.236; *P* = 0.034) and post-FMT (Spearman’s rho, 0.148; *P* = 0.003) samples. The relationship was validated by applying Procrustes analysis on the principal-coordinate analysis (PCoA) axes of the two biomes (sum of squares, 2.17 × 10^4^; Procrustes m2, 0.0471; correlation, 0.976; *P* = 0.001). Nonparametric correlations between fungal and bacterial taxa were examined, and two novel coexclusion relationships between *Candida* OTU2 (100% similarity to Candida albicans) and *Dorea* OTU18 (98.81% similarity to Dorea longicatena) and *Clostridium* XVIII OTU34 (98.41% similarity to Faecalibacillus intestinalis) were identified (Fig. 2C). Notably, these *Dorea* OTU18 and *Clostridium* XVIII OTU34 are enriched in donors (OTU18 LDA score of 3.76, *P* < 0.05; OTU34 LDA score of 3.90, *P* < 0.05) and in responders post-FMT (OTU18 LDA score of 3.86, *P* < 0.05; OTU34 LDA score of 3.62, *P* < 0.05) compared with baseline samples (Fig. 2D) but not in nonresponders following FMT.

### Donor microbiome engraftment persisted up to 6 months following FMT.

**FIG 3:**
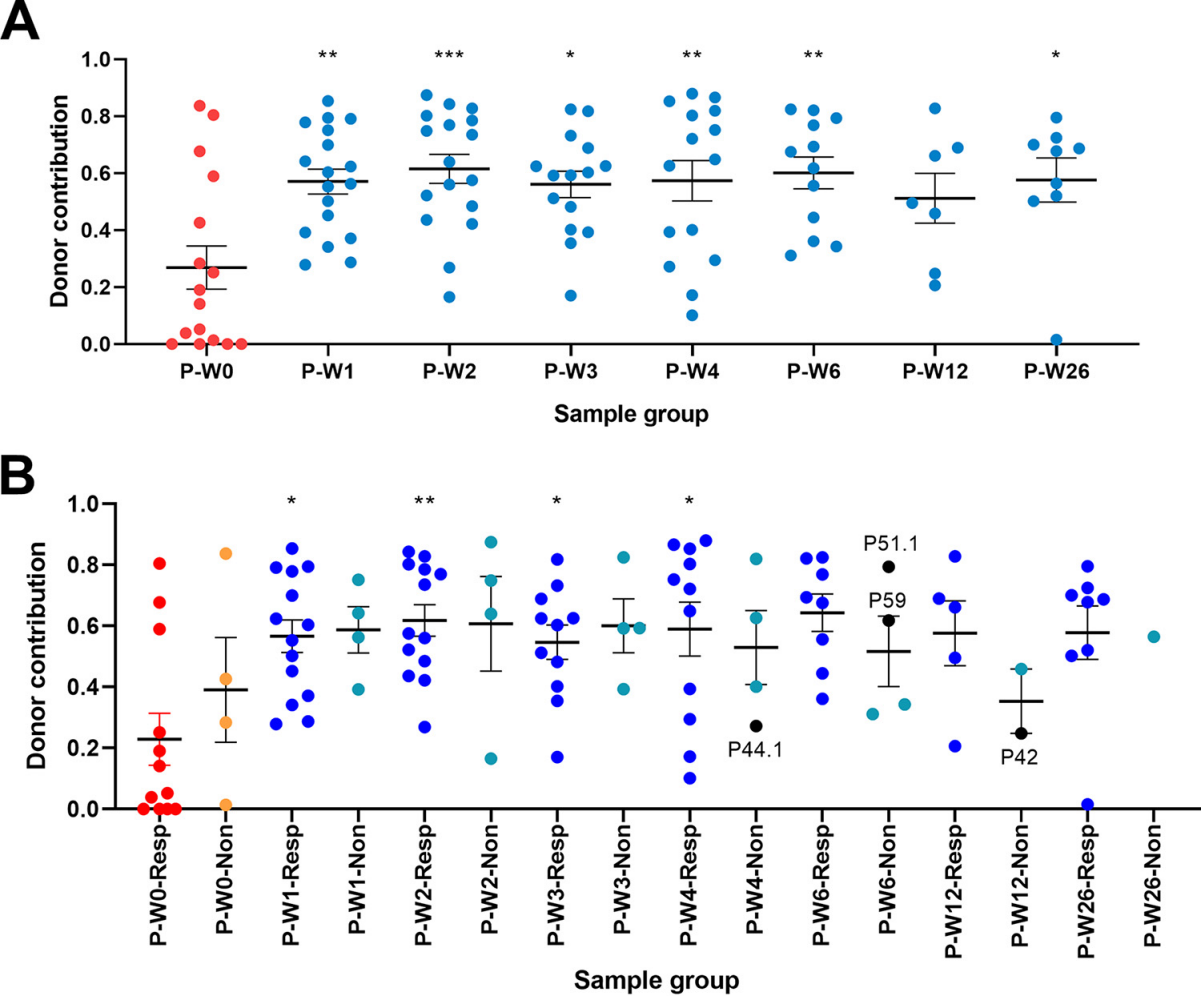
Donor contribution to the patient bacterial component of the microbiome. (A) Contribution was determined using SourceTracker with donor samples assigned as specific sources (one-to-one) to their matched patient samples (sinks). Significance was tested using ANOVA with Tukey’s multiple-comparison tests. Donor contribution to the baseline sample was significantly lower than the post-FMT samples with the exception of P-W12. (B) Donor contribution was stratified according to response (-Resp) or lack of response (-Non) to FMT. Contribution was determined using SourceTracker with donor samples assigned as specific sources to their matched patient samples (sinks). Patient samples at recurrence were labeled in black and with patient number. Significance was tested using ANOVA with Tukey’s multiple-comparison tests. Only baseline samples of responders (P-W0-Resp) were significantly different from other groups (denoted by asterisks above groups).*, *P* < 0.05; **, *P* < 0.01; ***, *P* < 0.001; ****, *P* < 0.0001.

Given the increased alpha diversity and compositional shifts in FMT recipients toward the donors, the levels and persistence of donor engraftment after FMT therapy were studied. The following two strategies were adopted: the first was a one-to-one strategy where the specific donors were matched to their recipient, and the second was an all-to-one strategy where we did not differentiate between donors. As expected, there was a significantly higher donor contribution following FMT across both strategies (Fig. 3A; see Fig. S7A in the supplemental material). While there was initial engraftment in both responders and nonresponders, it was more robust in responders and there was a reduction in donor species persistence seen in nonresponders from week 4 to 12 (Fig. 3B; Fig. S7B). However, this decrease in persistence may be the result of the lower number of samples in nonresponders and not a biological effect. Notably, high levels of donor contribution were seen up to 26 weeks (*P* < 0.001) following treatment in responders (Fig. 3; Fig. S7), suggesting stable long-term engraftment of bacteria even with single dose of oral lyophilized FMT. In contrast, fungal source tracking from donor to patient showed no significant increases from baseline to post-FMT (see Fig. S8 in the supplemental material).

10.1128/mSystems.00905-20.7 FIG S7 Donor contribution to the patient microbiome. (A) Contribution was determined using SourceTracker with all donor samples assigned as nonspecific sources (all-to-one) and all patient samples as sinks. Donor contribution to the baseline sample was significantly lower than that to the post-FMT samples. Significance was tested using ANOVA with Tukey’s multiple-comparison tests. (B) Donor contribution was stratified according to response (-Resp) or lack of response (-Non) to FMT. Contribution was determined using SourceTracker with all donor samples assigned as nonspecific sources and all patient samples as sinks. Significance was tested using ANOVA with Tukey’s multiple-comparison tests. Only baseline samples of responders (P-W0-Resp) were significantly different from other groups (denoted by asterisks above groups). *, *P* < 0.05; **, *P* < 0.01; ***, *P* < 0.001; ****, *P* < 0.0001. Download FIG S7, TIF file, 0.7 MB. Copyright © 2021 Haifer et al. 2021 Haifer et al. This content is distributed under the terms of the Creative Commons Attribution 4.0 International license.

10.1128/mSystems.00905-20.8 FIG S8 Donor contribution to the patient mycobiome. Contribution was determined using SourceTracker with donor samples assigned as specific sources (one-to-one) to their matched patient samples (sinks). Significance was tested using ANOVA with Tukey’s multiple-comparison tests. No significant differences were observed. Download FIG S8, TIF file, 0.3 MB. Copyright © 2021 Haifer et al. 2021 Haifer et al. This content is distributed under the terms of the Creative Commons Attribution 4.0 International license.

### Donor microbiome and FMT efficacy.

**FIG 4:**
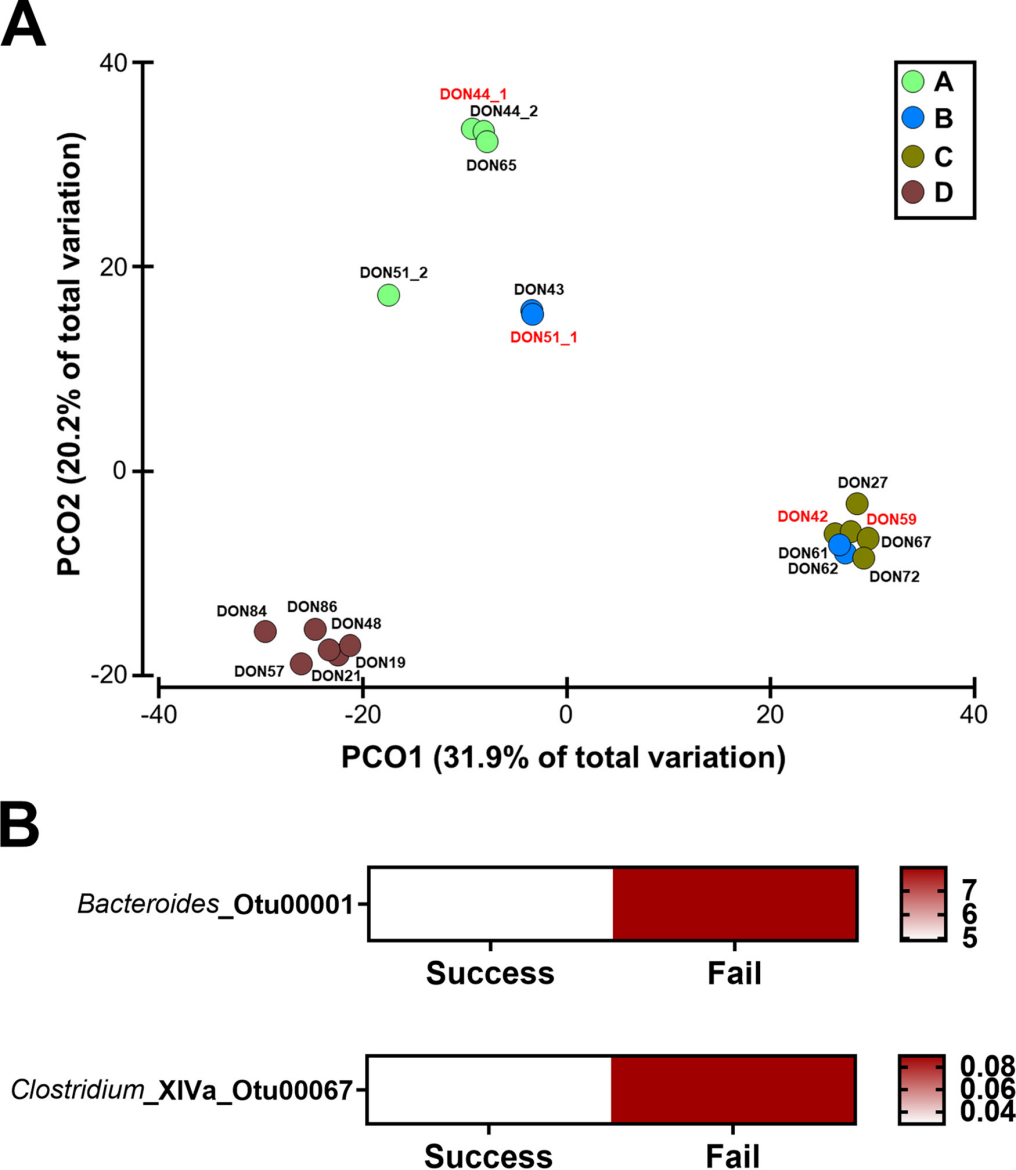
Bacterial communities in donor samples. (A) Principal-coordinate analysis of Bray-Curtis resemblance matrix generated from square-root-transformed relative abundances of bacterial OTUs. All donors (A, B, C, and D) were found to be significantly different from each other using pairwise PERMANOVA (*P* < 0.024 for all) except for donors B and C (PERMANOVA: *t* = 1.46, *P* = 0.054; ANOSIM: *r* = 0.288, *P* = 0.087). (B) Heatmap of mean relative abundance of bacterial OTUs found to be significantly different between donor samples that led to therapy success and those that led to therapy failure. Tests were performed using LEfSe and a cutoff LDA score of >3.5 and *P* value of <0.05 were applied.

FMT was derived from four individual unrelated and unmatched donors (Table 2). Across the whole cohort (*n* = 37), each donor provided FMT for at least 1 treatment failure. The most regular donor who provided 57% of the treatments had 2 episodes of treatment failure, of which both were in the setting of further antibiotic therapy. Given this result, donor microbial features associated with therapy outcomes were assessed. Species evenness and Shannon’s diversity were similar across donors and were relatively stable across time (Fig. 1A; Fig. S2B). Some variability in species richness was observed at an inter- but not intradonor level (Fig. S2A). These differences were not associated with treatment response. Donors clustered independently according to their composition (Fig. 4A) but did not appear to influence therapy outcome. Despite this finding, a common feature across donor FMTs contributing to treatment failure was significantly higher levels of taxa from *Bacteroides* sp. (98.81% sequence similarity to Bacteroides vulgatus) and *Clostridium* XIVa (LDA score of >3.5 and *P* < 0.05) (Fig. 4B), with the former likely reflecting lower levels of *Firmicutes* sp. in these samples.

**TABLE 2:** Baseline donor characteristics

Donor	Age (yrs)	Sex	No. of successful treatments (%)^*a*^
A	43	Male	2/3 (66.3)
B	53	Female	6/7 (85.7)
C	34	Female	6/7 (85.7)
D	54	Male	19/21 (91)

a One of the patients that was retreated had their second batch from a different donor.

### Primary CDI showed more pronounced microbiome shifts than recurrent CDI.

**FIG 5:**
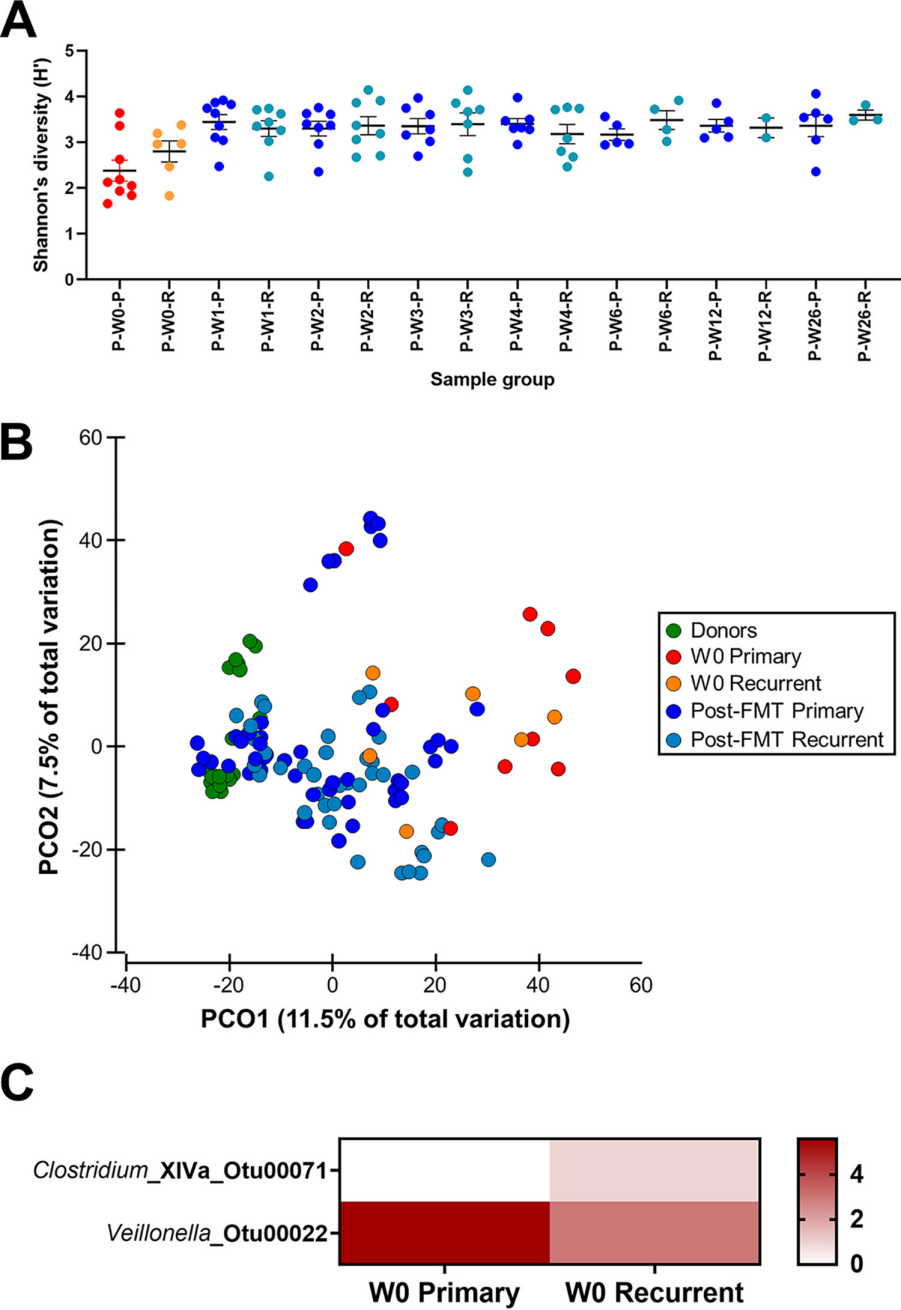
Differences in the bacterial communities between types of C. difficile infection. Only responders were included in this analysis. Severe C. difficile infections or those with persisting disease despite antibiotics were excluded due to low numbers leaving primary (-P) and recurrent (-R) infections. (A) Shannon’s diversity (H´) index across different sample groups. Significance was tested using ANOVA with Tukey’s multiple-comparison test, and only P-W0-P was found to be statistically significantly different from other post-FMT groups. (B) Principal-coordinate analysis of Bray-Curtis resemblance matrix generated from square-root-transformed relative abundances of bacterial OTUs. All patient subgroups (P-) were significantly different from the donors (DON) when tested using pairwise PERMANOVA (*P* < 0.046 for all). P-W0-P was consistently significantly different from all other post-FMT sample groups in primary CDI (*P* < 0.015 for all). This result could not be replicated in the patients with recurrent CDI. (C) Heatmap of mean relative abundance of bacterial OTUs found to be significantly different between patients with primary and recurrent CDI at baseline. Tests were performed using LEfSe, and a strict cutoff LDA score of >4 and *P* value of <0.05 were applied.

Primary and recurrent CDI had similar clearance rates post-FMT. The baseline microbiomes of these patients and the effects of FMT were compared, with the analysis limited to responders to avoid confounding by signatures related to lack of response (Fig. 5; see Fig. S9 in the supplemental material). Both patients with primary CDI and recurrent CDI responded similarly, with increases in bacterial alpha diversity measures and a shift in beta diversity toward the donor following FMT (Fig. 5; Fig. S9). However, the changes in bacterial community measures in primary CDI appeared to be more robust than those in recurrent CDI (*P* < 0.015 for all, PERMANOVA) (Fig. 5). Next, features that could discriminate between primary and recurrent CDI were assessed, and the relative abundance of a *Veillonella* taxon was identified as a strong marker of primary CDI (LDA score of >4 and *P* < 0.05) (Fig. 5C). This taxon was also found to be a marker in responders to FMT therapy (Fig. 1E). In contrast, on stratification of samples based on recurrent or primary CDI, no significant differences were observed across all mycobiome metrics (data not shown).

10.1128/mSystems.00905-20.9 FIG S9 Alpha diversity measures within the bacteriome across different types of C. difficile infection. Only responders were included in this analysis. Severe C. difficile infections or those not responsive to antibiotics were excluded due to low numbers leaving primary (-P) and recurrent (-R) infections. (A) Species richness (d) across different sample groups. Significance was tested using ANOVA with Tukey’s multiple-comparison test, and P-W0-P was found to be statistically significantly different from P-W1-P. No other comparisons were significant. (B) Species evenness (J´) across different sample groups. Significance was tested using ANOVA with Tukey’s multiple-comparison test, and only P-W0-P was found to be statistically significantly different from other post-FMT groups. Download FIG S9, TIF file, 0.6 MB. Copyright © 2021 Haifer et al. 2021 Haifer et al. This content is distributed under the terms of the Creative Commons Attribution 4.0 International license.

## DISCUSSION

In this prospective real-world cohort of consecutive patients with CDI, oral lyophilized FMT was safe and highly effective in treating both recurrent and primary CDI, with prolonged bacterial engraftment in patients who responded to therapy.

Our study showed that bacterial engraftment is successful in responders with a single treatment of orally administered lyophilized FMT, with microbial diversity increasing and composition shifting toward the donor profiles. Furthermore, microbial changes persisted out to 26 weeks following therapy. We did not see a significant shift in recipient composition in patients who did not respond to therapy. FMT success was characterized by a decrease in *Enterobacteriaceae* and an increase in *Faecalibacterium* sp. These findings are consistent with previous studies suggesting a beneficial role for short-chain fatty acid-producing species in the context of FMT (7).

In our cohort, the mycobiome signatures identified were less robust than those of bacterial signatures. We did however find that the recipient fungal richness and the presence of *Penicillium* sp. were associated with reduced therapeutic outcome. *Penicillium* sp. has been recognized as a prominent fungal element associated with CDI that may increase intestinal dysbiosis (14). This has been suggested to occur through the antibacterial compounds these species produce, which may affect the capacity of the microbiota to recover (15). Only one study has examined the impact of the mycobiome on therapeutic outcomes in CDI patients following FMT, which was administered via a nonoral route. In a previously published CDI cohort by Zuo et al., Candida albicans was shown to have a negative impact on patient response to naso-duodenal FMT therapy, and *Penicillium* species was a favorable finding (16). While we did not see a significant association between *Candida* species and a lack of response, we identified two novel and robust coexclusion relationships with Dorea longicatena and *Faecalibacillus intestinalis*, bacterial taxa that were enriched in donors and responders following therapy. Furthermore, the presence of one *Penicillium* taxon was associated with the lack of response to therapy. There are a few potential explanations for this discrepancy. The previous study assessed an Asian cohort of CDI, where a less severe CDI phenotype is sometimes seen (17). Dietary and geographical environmental differences might also explain the discordant *Penicillium* findings since these fungi are commonly found in foods.

It has been hypothesized that the lyophilization process can damage certain bacterial species and that an upper gastrointestinal (GI) route of administration can further reduce beneficial bacteria entering the colon (18). Whether the lack of shift in the nonresponder recipient microbiome is the cause for cases of treatment failure and a more intensive regime may improve clinical success rate are yet to be determined. In our study, one patient who had no clinical response to therapy and had low donor contribution at week 1 had clinical success with further oral FMT therapy from the same donor, supporting the notion of using repeated oral FMT in those CDI patients who do not have an initial or sustained clinical response to therapy.

Limitations of our study include the relatively small patient numbers from an uncontrolled cohort in a single expert center. While our findings are encouraging, a randomized controlled clinical trial is required to confirm the efficacy of oral FMT in primary CDI. A strength of the study was the frequent sample collection the cohort underwent, enabling longitudinal characterization of microbial dynamics, particularly fungal analysis out to 26 weeks, which has not been done in previous CDI cohorts. One notable characteristic of our patient cohorts is the younger age compared with those reported in the literature, and we believe this characteristic may reflect the mild-moderate disease included in this study.

In conclusion, our data confirmed that orally administered lyophilized FMT is effective in treating recurrent CDI and suggested that this form of therapy is safe and effective for primary CDI. Microbial engraftment was sustained in responders out to 6 months following therapy, with specific bacterial changes found to be associated with treatment outcomes. Novel coexclusion relationships were identified between *Candida* sp. and specific bacterial species associated with treatment efficacy, supporting the clinical relevance of transkingdom dynamics in CDI.

## MATERIALS AND METHODS

### Study design.

Consecutive adult patients with CDI treated with oral lyophilized FMT were prospectively enrolled in this “real world” cohort between 2015 and 2018 at a single center in Sydney, Australia. CDI was diagnosed by clinical symptoms and confirmed by detection of fecal CDI toxin and/or culture. Patients were treated with a single administration of 6 capsules (each 0.35-g lyophilized stools). No patients received FMT through another route. Treatment episodes were classified as either primary CDI for an initial episode of infection or as recurrent CDI for persistent or repeated disease following an appropriate course of antibiotics. Patients were followed weekly for 6 weeks and then monthly for 6 months to assess for symptom response, recurrence, and adverse events. Fecal samples were collected at baseline as well as weeks 1, 2, 3, 4, 6, 12, and 26 following FMT therapy for CDI toxin and culture. Sustained CDI cure was defined as a resolution of diarrhea in addition to loss of CDI toxin and/or culture with no symptom recurrence during the follow-up period. The study was approved by the Centre for Digestive Diseases Human Research Ethics Committee (CDD18/CO5). Written informed consent was obtained from all recruited study subjects.

### Fecal donors.

Fresh stool was obtained from four individual donors over a 2-year period. Donors were unmatched and unrelated and were screened according to previously published protocols (19).

### Lyophilized FMT production.

Donated stool was processed within 4 h of collection. Donor stool was homogenized with a cryoprotectant (trehalose and cysteine) and stored at −80°C for up to 2 weeks before being lyophilized. The freeze-dried product from each individual donor was then double encapsulated and stored at −80°C until dispensation. Each capsule contained 0.35 g of lyophilized stool.

### Sample collection and DNA extraction.

Fecal samples were collected from individual donors and study participants for gastrointestinal microbial community profiling. Twenty-three patients (62.2% of patient cohort) and 4 donors (100% of donors) provided a total of 166 fecal samples from baseline as well as weeks 1, 2, 3, 4, 6, 12, and 26 following FMT therapy. All samples were homogenized and then stored at −80°C until nucleic acid extraction. DNA was extracted using the QIAamp PowerFecal DNA kit (Qiagen, Chadstone, VIC, Australia) according to the manufacturer’s instructions.

### 16S rRNA amplicon sequencing.

The V4 region of the 16S rRNA gene was amplified using the Kapa HiFi HotStart ReadyMix (95°C for 3 min; 25 cycles of 95°C for 30 s, 55°C for 30 s, and 72°C for 30 s; followed by a final step of 72°C for 5 min) and the Earth microbiome primers (515F and 806R) (20) as previously described (21). Indices and Illumina sequencing adapters were attached using the Nextera XT index kit, and sequencing was performed with Illumina MiSeq 2 × 250-bp chemistry at the Ramaciotti Centre for Genomics. Three negative-control samples (extraction kit reagents) were included as part of the sequencing run. Raw reads were analyzed using mothur v1.42.3, (22, 23) with Silva SEED 16S rRNA reference (v123) alignment, clustering with opticlust method, and classification using RDP v16 (3%). The resulting data matrix was subsampled (read depth, 25,287 clean reads/sample) and used for statistical analysis.

### ITS region amplicon sequencing.

The fungal internal transcribed spacer (ITS) region was amplified using the primers fITS7 and ITS4 (24, 25). Raw reads were analyzed using mothur v1.42.3. Reads were clustered by abundance (method, agc) and classified using the UNITE v6 database (cutoff, 0.05). The same three extraction controls as above were used for the fungal ITS sequencing, and minimal amplification was observed (see Fig. S1 in the supplemental material). The resulting data matrix was used for statistical analysis (mean read depth, 47,906 clean reads/sample).

### Statistical analysis.

Calculation of alpha diversity measures, correlation of resemblance matrices (RELATE), principal-coordinate analysis (PCoA), analysis of similarities (ANOSIM), and permutational multivariate ANOVA (PERMANOVA) were performed using Primer-E v6. Per taxon analyses were conducted using LEfSe (26). Source tracking of microbial taxa was performed using SourceTracker (27) within the Metagenomics for Environmental Microbiology Galaxy framework (28). Procrustes and protest analyses were performed using the R package “vegan,” and nonparametric correlation analyses were performed using the framework outlined in Reshef et al. (29). All additional statistical analyses were performed using GraphPad Prism v8.

### Data availability.

The data sets generated during the current study are available in the European Nucleotide Archive repository under the accession numbers PRJEB37800 (16S) and PRJEB37810 (ITS).
