# Supplementary figures and images for: Long-Term Bacterial and Fungal Dynamics following Oral Lyophilized Fecal Microbiota Transplantation in Clostridioides difficile Infection
Source: mSystems. 2021 Feb 2;6(1):e00905-20. doi: 10.1128/mSystems.00905-20 (PMC7857531; doi:10.1128/mSystems.00905-20)

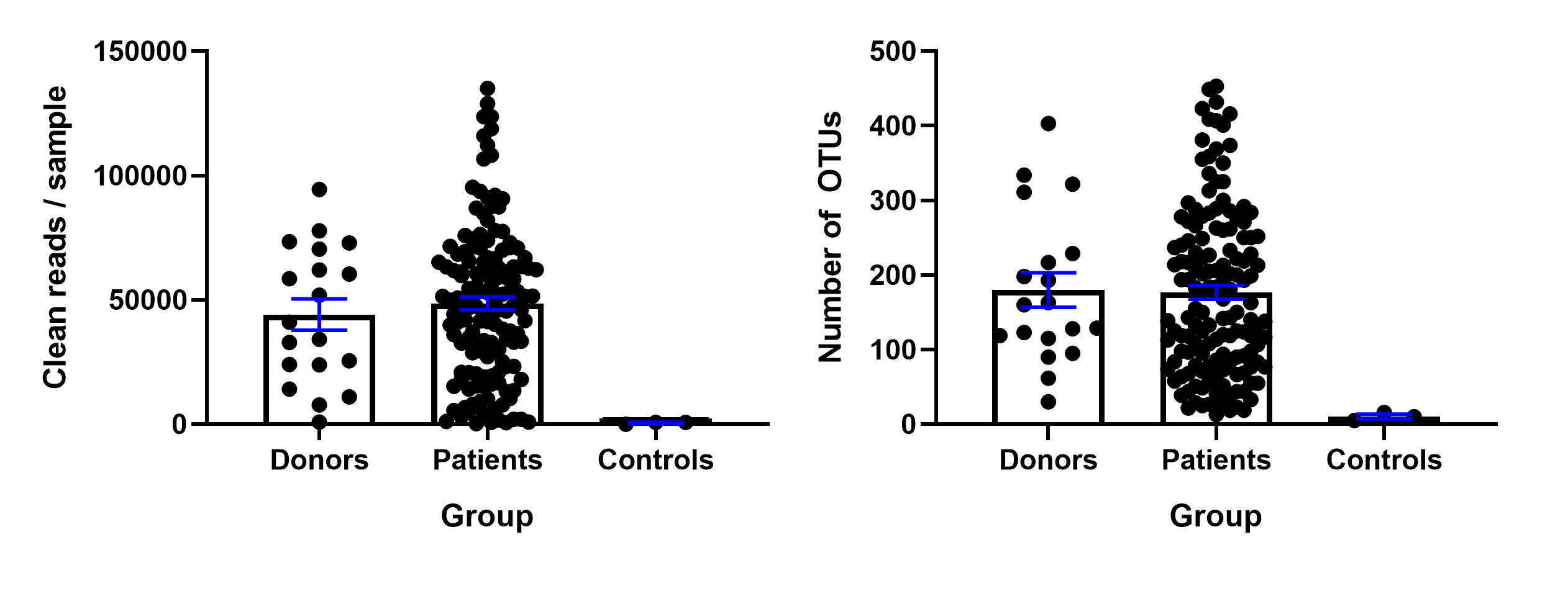

Supplement: FIG S1 [file msystems.00905-20-sf001.tif]

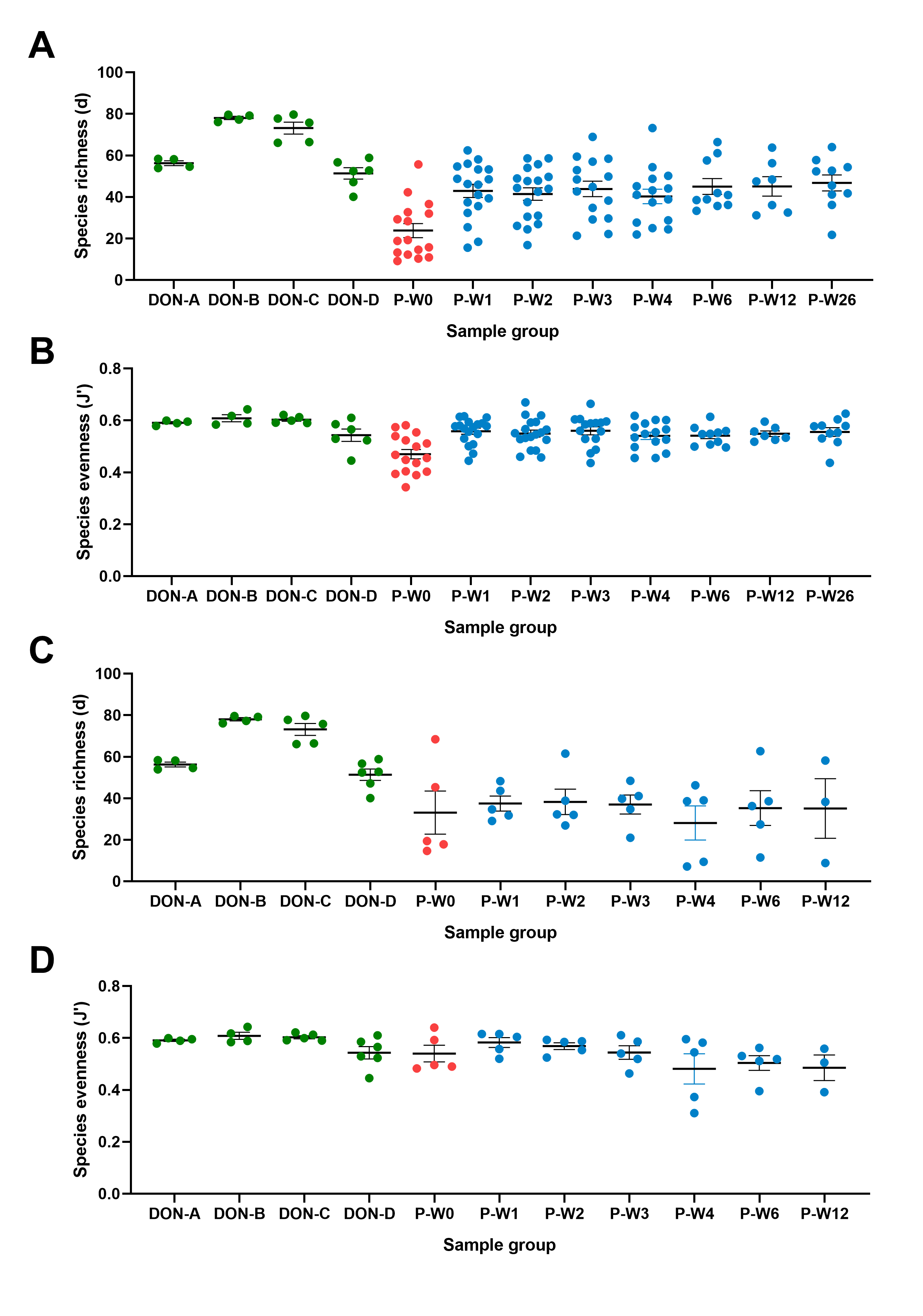

Supplement: FIG S2 [file msystems.00905-20-sf002.tif]

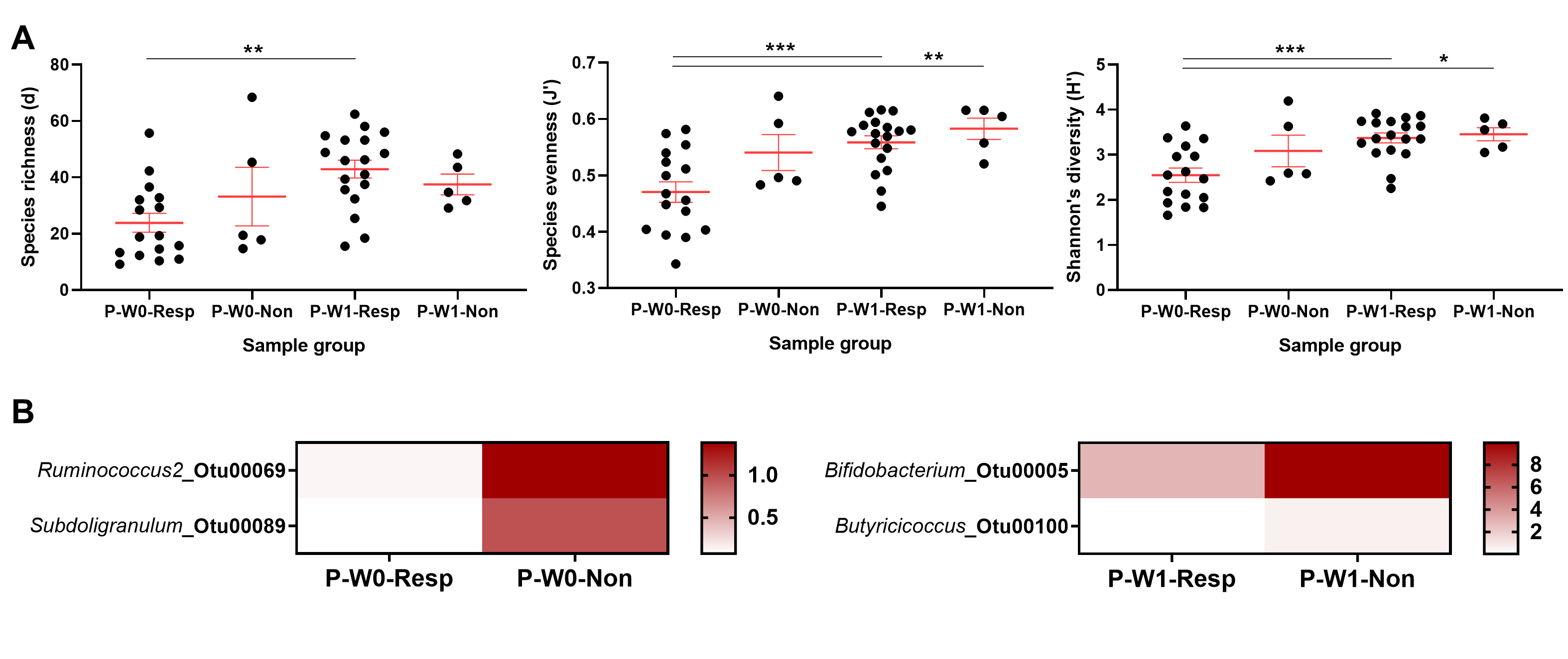

Supplement: FIG S3 [file msystems.00905-20-sf003.tif]

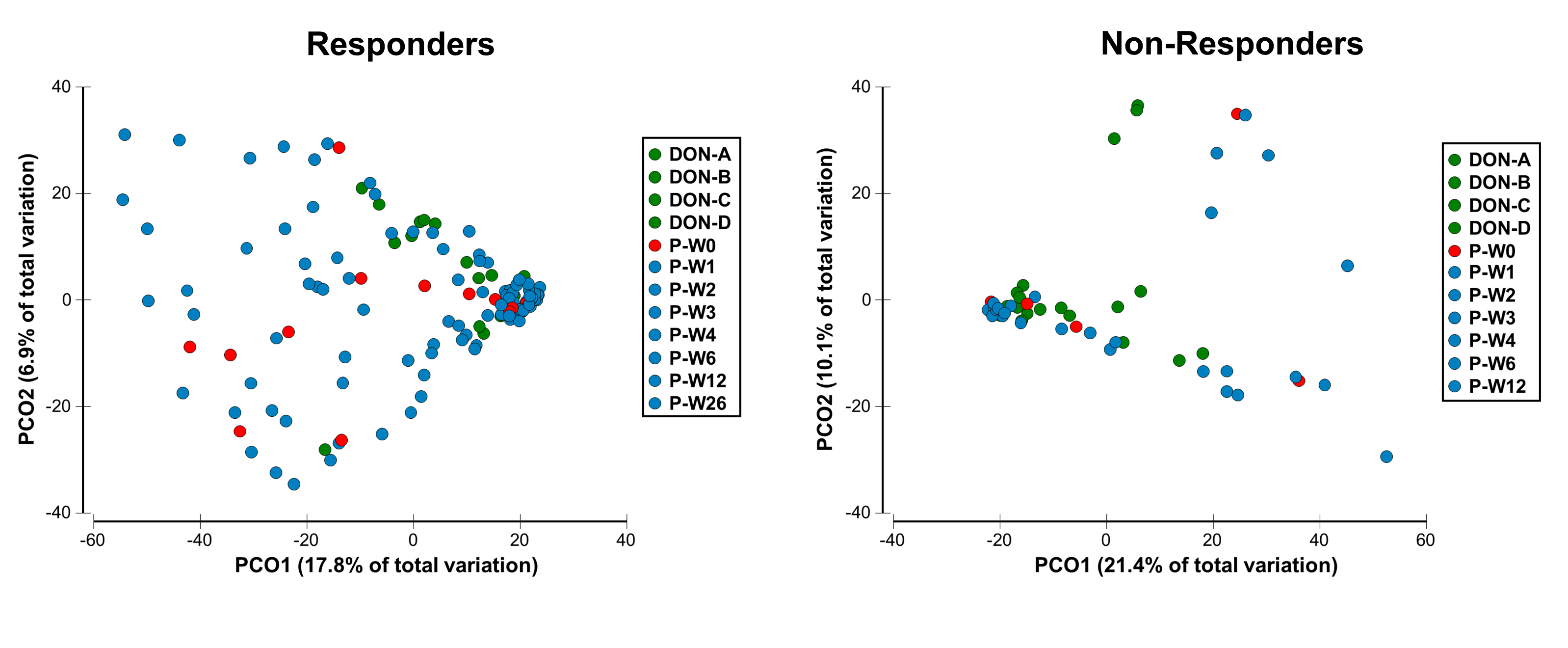

Supplement: FIG S4 [file msystems.00905-20-sf004.tif]

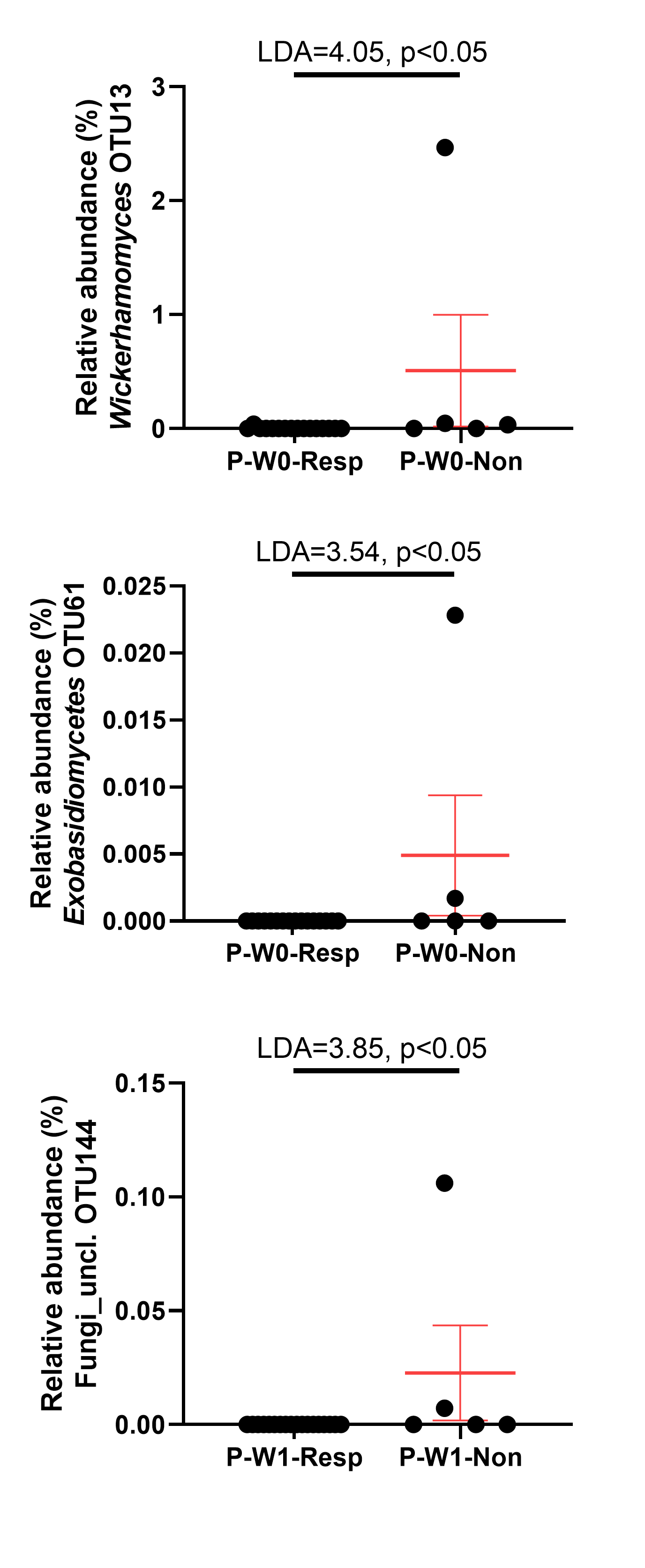

Supplement: FIG S5 [file msystems.00905-20-sf005.tif]

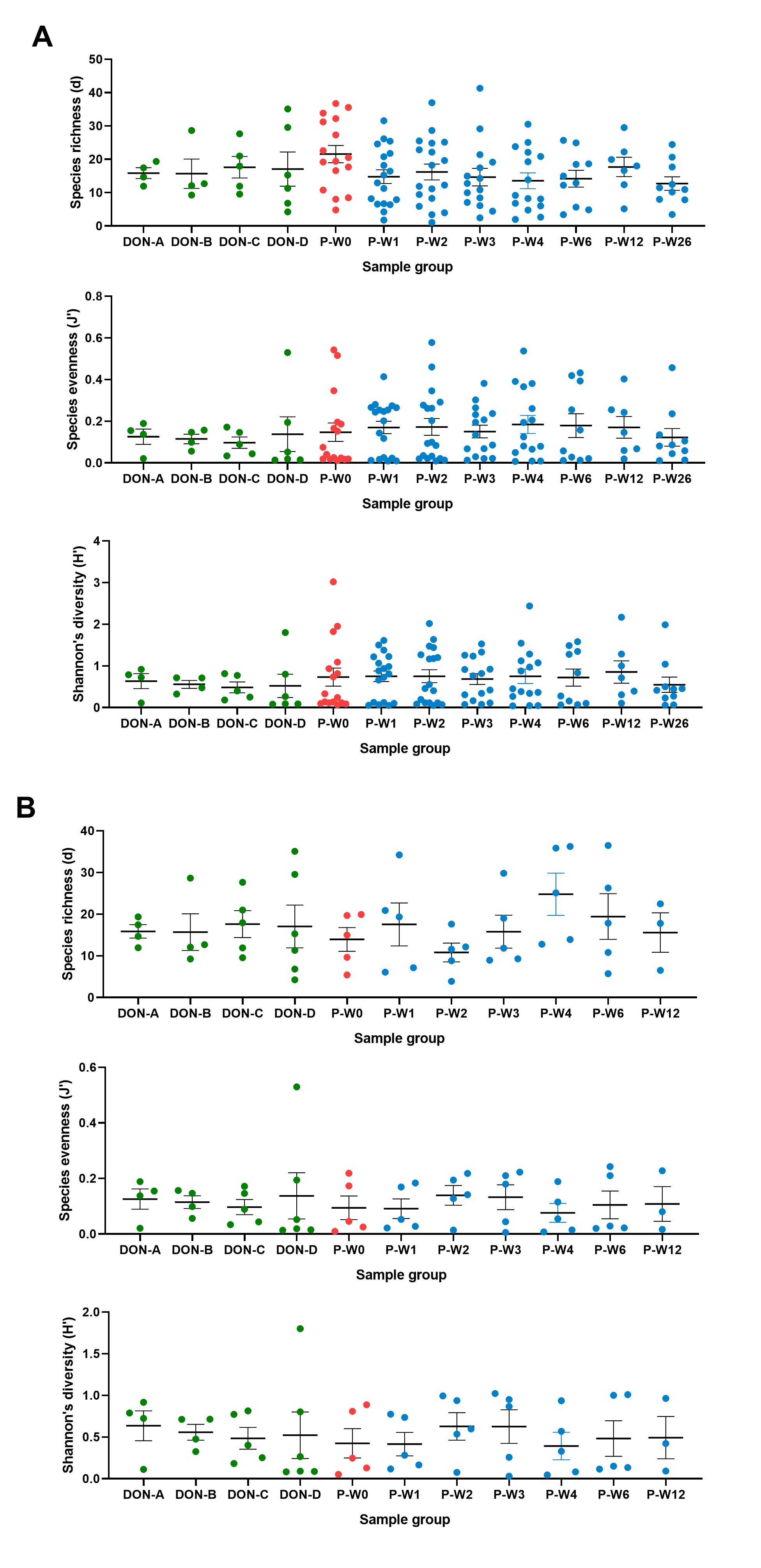

Supplement: FIG S6 [file msystems.00905-20-sf006.tif]

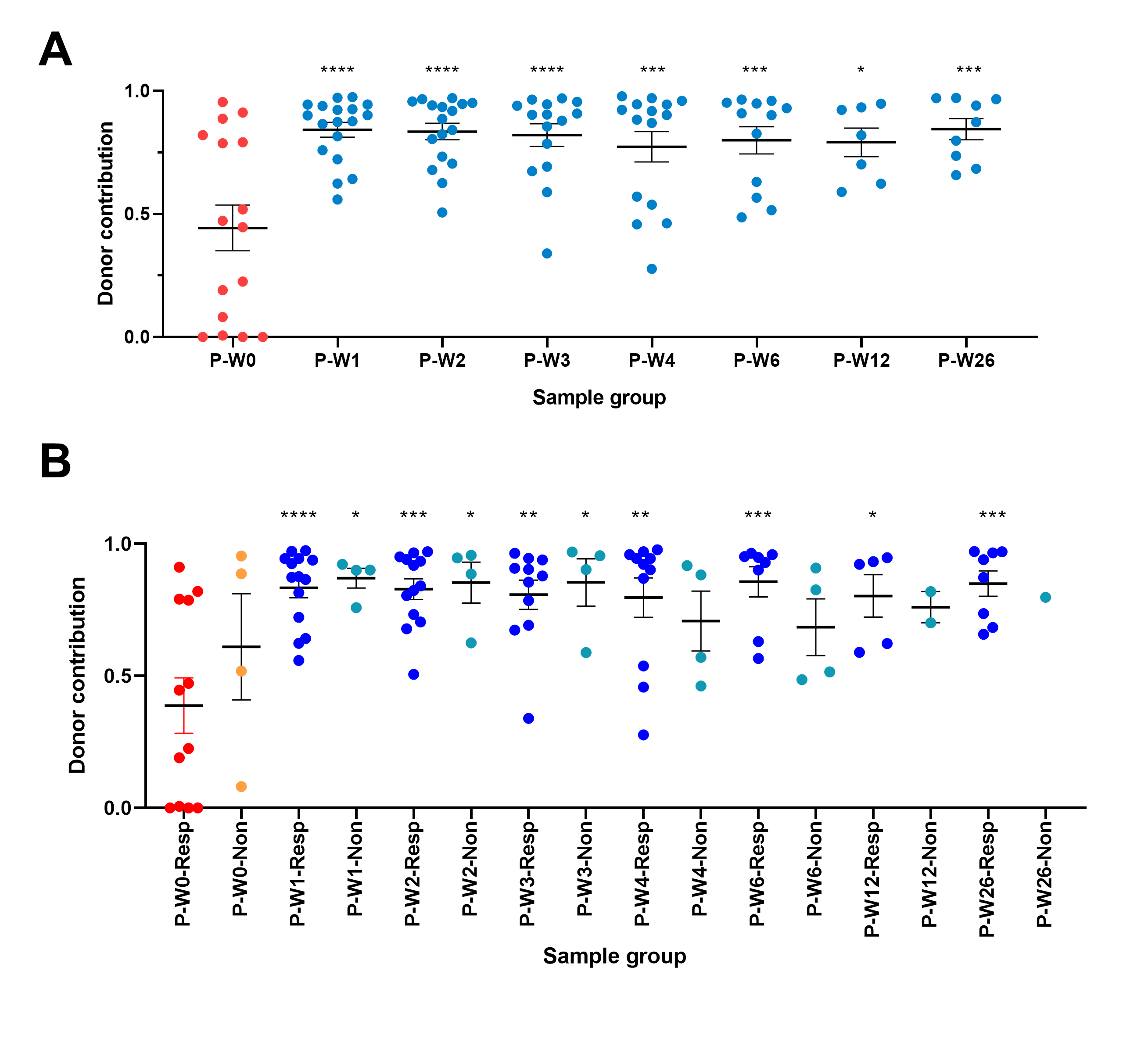

Supplement: FIG S7 [file msystems.00905-20-sf007.tif]

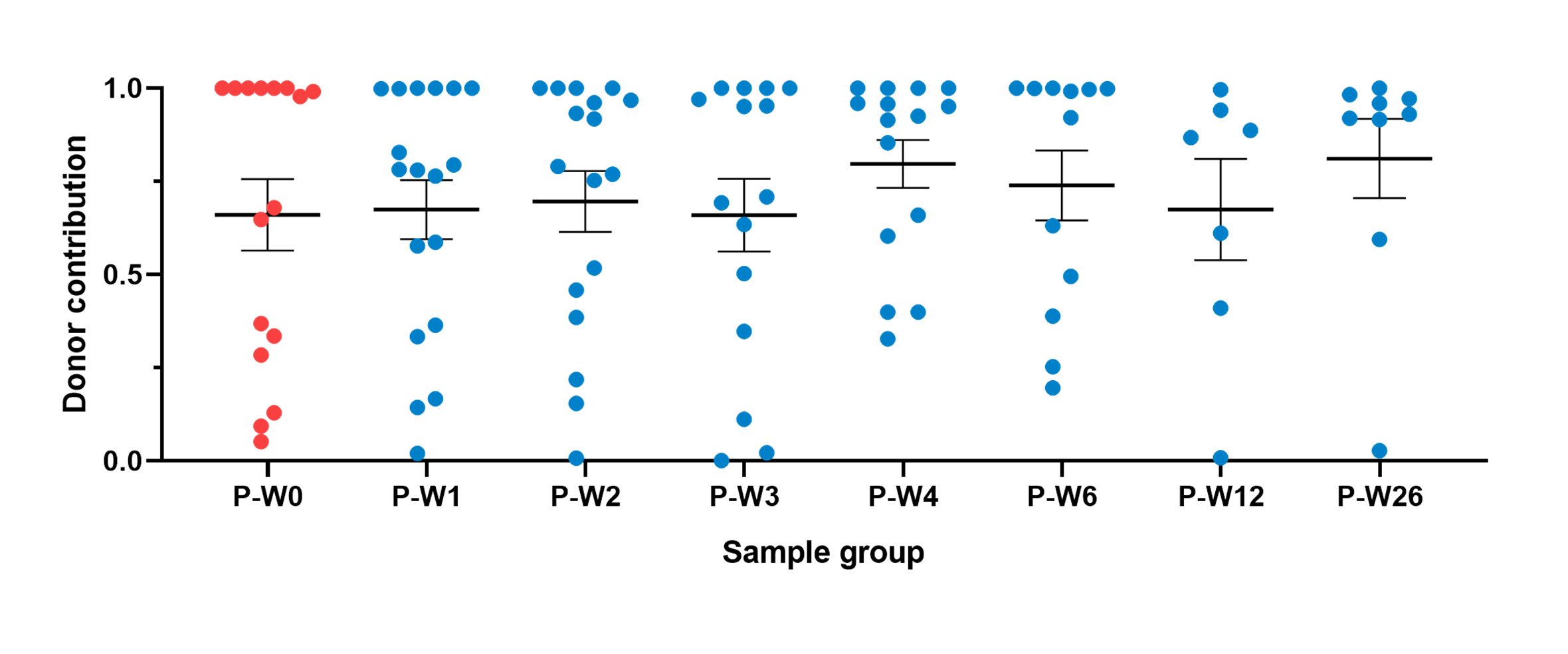

Supplement: FIG S8 [file msystems.00905-20-sf008.tif]

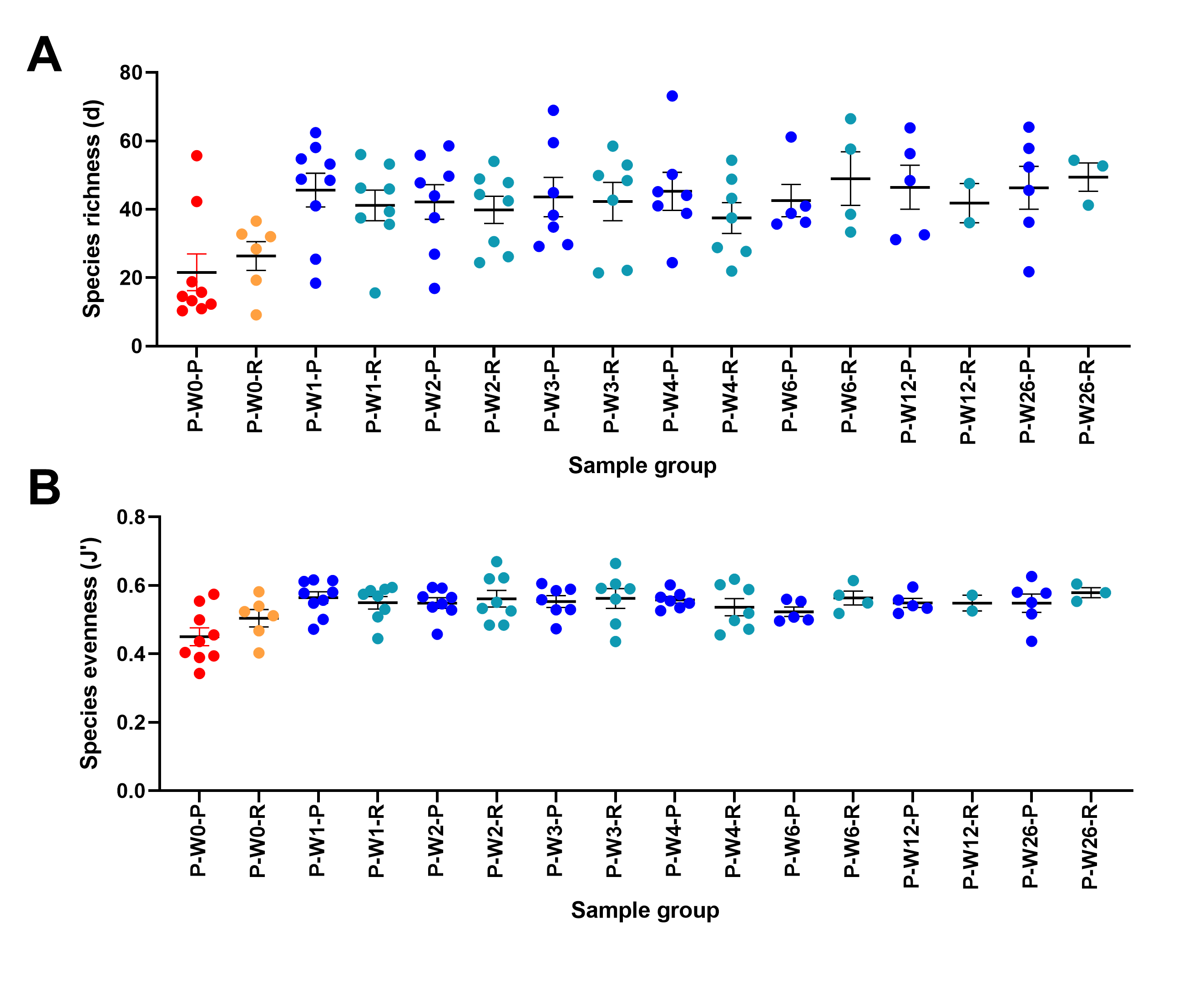

Supplement: FIG S9 [file msystems.00905-20-sf009.tif]
